# Matching tRNA modifications in humans to their known and predicted enzymes

**DOI:** 10.1093/nar/gkz011

**Published:** 2019-01-30

**Authors:** Valérie de Crécy-Lagard, Pietro Boccaletto, Carl G Mangleburg, Puneet Sharma, Todd M Lowe, Sebastian A Leidel, Janusz M Bujnicki

**Affiliations:** 1 Department of Microbiology and Cell Sciences, University of Florida, Gainesville, FL 32611, USA; 2 Cancer and Genetic Institute, University of Florida, Gainesville, FL 32611, USA; 3 Laboratory of Bioinformatics and Protein Engineering, International Institute of Molecular and Cell Biology, ul. Trojdena 4, 02-109 Warsaw, Poland; 4 Max Planck Research Group for RNA Biology, Max Planck Institute for Molecular Biomedicine, 48149 Muenster, Germany; 5 Cells-in-Motion Cluster of Excellence, University of Muenster, 48149 Muenster, Germany; 6 Department of Biomolecular Engineering, University of California, Santa Cruz, Santa Cruz, CA 95064, USA; 7 Research Group for RNA Biochemistry, Institute of Chemistry and Biochemistry, University of Bern, 3012 Bern, Switzerland; 8 Institute of Molecular Biology and Biotechnology, Faculty of Biology, Adam Mickiewicz University, ul. Umultowska 89, 61-614 Poznań, Poland

## Abstract

tRNA are post-transcriptionally modified by chemical modifications that affect all aspects of tRNA biology. An increasing number of mutations underlying human genetic diseases map to genes encoding for tRNA modification enzymes. However, our knowledge on human tRNA-modification genes remains fragmentary and the most comprehensive RNA modification database currently contains information on approximately 20% of human cytosolic tRNAs, primarily based on biochemical studies. Recent high-throughput methods such as DM-tRNA-seq now allow annotation of a majority of tRNAs for six specific base modifications. Furthermore, we identified large gaps in knowledge when we predicted all cytosolic and mitochondrial human tRNA modification genes. Only 48% of the candidate cytosolic tRNA modification enzymes have been experimentally validated in mammals (either directly or in a heterologous system). Approximately 23% of the modification genes (cytosolic and mitochondrial combined) remain unknown. We discuss these ‘unidentified enzymes’ cases in detail and propose candidates whenever possible. Finally, tissue-specific expression analysis shows that modification genes are highly expressed in proliferative tissues like testis and transformed cells, but scarcely in differentiated tissues, with the exception of the cerebellum. Our work provides a comprehensive up to date compilation of human tRNA modifications and their enzymes that can be used as a resource for further studies.

## INTRODUCTION

The acquisition of post-transcriptional chemical modifications is an essential part of the maturation process required to generate functional tRNA molecules (1). Modifications have different roles in controlling stability, folding and decoding properties of tRNAs and can be determinants or anti-determinants for other components of the translation apparatus like e.g. aminoacyl-tRNA synthetases (2,3). In addition, tRNA modifications can be recognition elements of ribonucleases (4), leading to the generation of tRNA fragments that affect multiple cellular processes (5).

However, very few modifications such as m^1^G37, Ψ55 or t^6^A37 are present at a specific position of a particular tRNA in (almost) all known organisms. Most of them are specific to particular taxons, from species to kingdoms. For example, lysidine (k^2^C34) is a hallmark of bacteria (6), while archaeosine (G^+^15) is only found in archaea (7). Depending on the organism, the total number of genes encoding tRNA modification enzymes varies between as little as eleven in some obligate symbionts (8) to around an estimated hundred in humans of which 50 are currently represented in MODOMICS (9).

The near complete sets of tRNA modification genes are currently available for only one organism per domain of life: *Saccharomyces cerevisiae* for eukarya, where only one gene required for the formation of ncm^5^U out of cm^5^U is missing (10), *Escherichia coli* for bacteria where only the genes for ho^5^U34 and Acp^3^U47-formation remain unidentified and *Haloferax volcanii* for archaea where a handful of genes are missing (1). Beyond these three organisms, the annotation of tRNA modification genes remains scarce, because of several issues: First, RNA modification enzymes are often part of large multifunctional protein families such as the Rossmann Fold Methyltransferase (RFM) superfamily, which can act on other substrates than RNA (11). For example, some RNA methyltransferases are closely related to protein methyltransferases or DNA methyltransferases (11). Second, closely related members of orthologous families often introduce a similar chemical modification, but in different RNAs and at different positions. For example, members of the TrmFO family methylate tRNA or rRNA depending on the organism (12). Third, related enzymes can generate chemically distinct modifications. For example closely related Radical-SAM enzymes introduce methyl groups at different positions of nucleosides like in m^2^A or m^8^A (13). Finally, the same chemical modification, in particular methyl groups, can be introduced by proteins that are dissimilar (14) or even evolutionarily unrelated having arisen through non-orthologous gene displacements (15). For example, the formation of the universal m^1^G37 is catalyzed by TrmD and Trm5, two enzymes of completely different evolutionary origins in bacteria and in eukarya/archaea (16). The combination of these factors has made it difficult to identify enzymes responsible for many tRNA modifications and hence to determine the function of those tRNA modifications in many species including humans.

Recently, an increasing number of mutations causing genetic diseases have been mapped to human genes encoding tRNA modification enzymes (see (17–22) and Table 1), making a comprehensive list of these genes highly desirable. However, to our knowledge, no complete compilation of modifications found in both cytosolic and mitochondrial human tRNAs with their corresponding predicted or validated modification enzymes is available. For mitochondria, the best approximation is a recent list of modifications of bovine tRNAs and the predicted enzymes (23), which has been extrapolated for human tRNAs (23,24). A prediction of human tRNA methyltransferases, based on the known yeast enzymes was performed more than five years ago (25) and was recently extended to homologs of the other yeast RNA modification genes (26). Surveys of specific enzyme families such as the human m^5^C methyltransferases (27) or pseudouridine synthases (28) that target tRNA molecules have listed the known and missing genes for these specific modifications. The goal of our analysis was to compile a comprehensive list of known and predicted tRNA modifications in *Homo sapiens* with genes implicated in their biosynthesis. This analysis allowed for the identification of the remaining gaps of knowledge in the field of human tRNA modifications and will help to guide future experiments. Furthermore, we have used publicly available datasets in order to determine the expression profiles and proteomic evidence of known and predicted modification enzymes. Our work will facilitate access to the current knowledge on human tRNA modification enzymes for a wider community of biologists.

**Table 1.** Known and predicted tRNA modification genes that have been linked to human diseases

Modification	Gene	Disease	Cyto. Pheno.	Mito. Pheno.	Article
xG	**THG1L**	Microcephaly, developmental delay, nephrotic defect	+	+	(110,175,176)
m^1^G	**TRMT10A**	Diabetes, intellectual disabilities, microcephaly, developmental defects	+		(111,143,177–180)
ac^4^C	**NAT10**	Cancer	+		(112,181,182)
ac^4^C	**THUMPD1**	Cancer	+		(113)
Gm	**TARBP1**	Cancer	+		(114,115)
D	**DUS2**	Cancer	+	+	(116)
Y	**PUS1**	Mitochondrial myopathy and sideroblastic anemia (MLASA)	+	+	(117,183,184)
m^3^C	**METTL6**	Cancer	+		(118,119)
I	**ADAT3**	Intellectual disabilities, microcephaly	+		(120,185–187)
m^5^C	**NSUN2**	Intellectual disabilities, developmental delay, reduced fertility, cancer	+		(121,170–172,189,229–232)
C_m_,U_m_,G_m_, f^5^C_m_, hm^5^C_m_, mcm^5^U_m_	**FTSJ1**	Intellectual disabilities	+		(122,123,188)
C_m_, G_m_,f^5^C_m_, hm^5^C_m_	**WDR6**	Cancer	+		(124)
Q	**QTRT1**	Cancer	+		(125)
cm^5^U, ncm^5^U, mcm^5^U, mcm^5^s2U	**ELP1**	Familial dysautonomia, cancer	+		(126,127,190)
cm^5^U, ncm^5^U, mcm^5^U, mcm^5^s^2^U	**ELP3**	Familial dysautonomia, Charcot–Marie–Tooth disease (CMT), cancer, amyotrophic lateral sclerosis (ALS)	+		(127,130,191,192)
cm^5^U, ncm^5^U, mcm^5^U, mcm^5^s^2^U	**ELP4**	Autism spectrum disorder, intellectual disabilities	+		(128)
cm^5^U, ncm^5^U, mcm^5^U, mcm^5^s^2^U	**ELP5**	Cancer, diabetes	+		(129,193,194)
s^2^U, mcm^5^s^2^U	**CTU1**	Cancer	+		(127,130,131)
s^2^U, mcm^5^s^2^U	**CTU2**	Microcephaly, nephrotic defect, cancer	+		(127,130,132,133)
s^2^U, mcm^5^s^2^U	MOCS3*	Molybdenum cofactor deficiency			
s^2^U, mcm^5^s^2^U	MPST*	Mercaptolactate-cysteine disulfiduria (MCDU), intellectual disabilities			
s^2^U, mcm^5^s^2^U	NFS1*	Friedreich ataxia			
s^2^U, mcm^5^s^2^U	SERGEF*	Hereditary deafness, artheriosclerosis			
s^2^U, mcm^5^s^2^U	CIAO1*	Hereditary paraganglioma-pheochromocytoma syndromes, retinitis pigmentosa			
s^2^U, mcm^5^s^2^U	NUBP1*	Cancer			
s^2^U, mcm^5^s^2^U	ISCU*	Myopathy with lactic acidosis, Friedreich ataxia			
I	**ADAT1**	Coronary artery disease	+		(134)
m^1^G, m^1^I	**TRMT5**	Failure to thrive, hypertrophic cardiomyopathy, exercise intolerance	+	+	(135,136)
o2Yw, yW	**TRMT12**	Cancer			(137,138)
o2Yw, yW	**LCMT2**	Cancer	+		(139)
t^6^A	**YRDC**	Cancer	+		(140)
t^6^A	**OSGEP**	Galloway-Mowat syndrome, microcephaly, nephrotic defects	+		(18,141,195–197)
t^6^A	**TP53RK**	Galloway-Mowat syndrome, microcephaly, nephrotic defects, cancer	+		(141,142,195,196)
t^6^A	**TPRKB**	Galloway-Mowat syndrome, microcephaly, nephrotic defects	+		(141,195,196)
t^6^A	**LAGE3**	Galloway-Mowat syndrome, microcephaly, nephrotic defects	+		(141,195,196)
ms^2^t^6^A	**CDKAL1**	Diabetes, microcephaly, cancer	+		(144,198,199)
m^5^C	**TRDMT1**	Metabolism, cancer	+		(145,146)
Y	**PUS3**	Intellectual disabilities	+		(147,148)
Um	**TRMT44**	Partial Epilepsy with Pericentral Spikes (PEPS)	+		(149)
m^7^G	**METTL1**	Multiple sclerosis, cancer	+		(150,200,201)
m^7^G	**WDR4**	Microcephaly, cancer, nephrotic defects, developmental defects	+		(151,202–204)
m^5^U	**TRMT2A**	Cancer	+		(152)
Y	**PUS10**	Autoimmune diseases, intellectual disabilities	+		(153,205,206)
m^1^A	**TRMT6**	Cancer	+		(139,154,155)
m^1^A	**TRMT61A**	Cancer	+		(139,154,155)
m^5^C	NSUN6*	Cancer	+		(156)
m^1^G,m^1^A	**TRMT10C**	Lactic acidosis, hypotonia, feeding difficulties, deafness		+	(157,158)
m^1^G,m^1^A	**HSD17B10**	Neurodegeneration, cardiomyopathy		+	(158,207,208)
m^2,2^G	**TRMT1**	Intellectual disabilities, microcephaly	+	+	(159,209,210)
f^5^C	**NSUN3**	Cancer		+	(160,161)
tm^5^U	**GTPBP3**	Mitochondrial encephalopathy, lactic acidosis, and stroke-like episodes (MELAS), non-syndromic hearing loss	+		(162,163,211–213)
tm^5^U	**MTO1**	Lactic acidosis, cardiomyopathy, encephalopathy, non-syndromic hearing loss, cancer, myoclonus epilepsy associated with ragged-red fibers (MERRF)	+		(162–164,214–217)
tm^5^s^2^U	**TRMU**	Leigh syndrome, hepatopathy associated with hyperlactatemia, non-syndromic hearing loss	+		(165,218–222)
t6A	**OSGEPL1**	Cancer, MERRF		+	(166,167)
i6A	**TRIT1**	Microcephaly, developmental delay, epilepsy, cancer	+	+	(168,223–225)
ms2i6A	**CDK5RAP1**	Cancer, type II diabetes, vitiligo		+	(169,226–228)
m^1^A	**TRMT61B**	Cancer, Alzheimer's disease		+	(173,174)

*Disease likely caused by defects other than loss of tRNA modification.

## MATERIALS AND METHODS

The set of human isoacceptor tRNAs (i.e. tRNAs that are acylated with the same amino acid regardless of the anticodon sequence) was extracted from the Genomic tRNA Database (GtRNAdb): http://gtrnadb.ucsc.edu/ (29) and is summarized here: http://gtrnadb.ucsc.edu/genomes/eukaryota/Hsapi19/

All modifications present in the sequences of cytosolic and mitochondrial tRNA of human (*H. sapiens*), cow (*Bos taurus*), rat (*Rattus norvegicus*), and mouse (*Mus musculus*) were extracted from the MODOMICS database of RNA modification pathways (http://modomics.genesilico.pl/) (9). This provided a first list that was then updated with one modification from the literature (m^5^C34 in Leu-CAA-tRNA) and several human modifications detected with novel tRNAseq methods (30) (m^3^C20 in Met-CAU-tRNA and m^3^C47 in Leu-CAG-tRNA and most Ser-tRNAs, m^1^A16 in mito-Arg-TCG-tRNA and m^3^C32 in mito-Thr-UGU-tRNA and mito-Ser-UGA) that were missing from MODOMICS. The MODOMICS database was updated accordingly.

High-throughput tRNA-seq modification data was derived from published study data sets (30–32). The protein and literature mining tools of NCBI (33) as well as the Uniprot resource and Id/Mapping tools (34) were used to gather data. Gene names were gathered from the HUGO Gene Nomenclature Committee (https://www.genenames.org) (35). Protein interaction data was derived from BioGrid (36) and the predicted mitochondrial localization from MitoCarta (https://www.broadinstitute.org/files/shared/metabolism/mitocarta/human.mitocarta2.0.html) (37).

Human co-expression data was extracted from the Search-based Exploration of Expression Compendium (SEEK) database (http://seek.princeton.edu/index.jsp) (38). Phylogenetic trees for specific protein families were extracted from PhylomeDB (http://phylomedb.org) (39). For gene expression analyses, RNAseq data was obtained from the GTEx portal (www.gtexportal.org; GTEx_Analysis_2016-01-15_v7_RNASeQCv1.1.8_gene_tpm.gct.gz) on 30/04/2018. For each gene the transcript with the highest expression levels was selected for each tissue. Subsequently, relative expression levels were calculated and plotted as a heatmap using the heatmap.2 function in R. Tissues included in the analysis were selected to provide a general physiological overview. Hierarchical clustering of genes was performed according to similarity of expression profile using Ward's method (40). For tissue-specific proteomics evidence, we used the human proteome map (http://www.humanproteomemap.org/) using the default settings (41). Proteins that were not detected in any tissue were manually removed.

## RESULTS AND DISCUSSION

### Compiling all mammalian cytosolic tRNA modifications

As a first step to predict the complete set of modification enzymes, we sought to list the nature and positions of all chemical modifications that have been identified in human cytosolic tRNAs. This task is not trivial as the set of human tRNAs used in decoding is very complex (see (42) for a recent review). Indeed, not all tRNA sequences encoded in the human genome are expressed in common cell-lines (43,44). Based on the loss of canonical secondary structure, mutations at highly conserved positions, or positioning in transcriptional silent chromosomal regions, some candidate tRNA genes are likely tRNA-derived Short Interspersed Nuclear Elements or pseudogenes, and others may have non-canonical functions outside of translation (5,45,46). Therefore, additional filtering criteria are needed to select a list of tRNAs that *most likely* decode mRNAs in the human cytosol. An updated set of ‘high confidence’ human tRNAs has been generated by tRNAscan-SE 2.0 (Chan, Lin and Lowe, unpublished data) and is available in the GtRNAdb (29). This list of over 400 tRNA genes contains 47 distinct isoacceptors families (including tRNA^Sec^, the tRNA for selenocysteine insertion).

A first set of biochemically-determined tRNA isoacceptor sequences that include chemical modifications in at least one mammal was extracted from the MODOMICS database at the time of the initial analysis (30 May 2017). Furthermore, partial information is available for modifications at specific positions such as wobble uridine (U34). It is known that 5-carbamoylmethyluridine (ncm^5^U) is found in Val-UAC-tRNA, 5-methoxycarbonylmethyl-2-thiouridine (mcm^5^s^2^U) in Arg-UCU-tRNA and 5-methoxycarbonyl-hydroxymethyluridine (mchm^5^U) in Gly-UCC-tRNA (47). Since the modifications of U34 in human Arg-UCU-tRNA and Gly-UCC-tRNA differ from those in the corresponding yeast tRNA, it is difficult to predict the nature of most of the U34 modifications in humans (48). Finally, a large fraction of the RNA sequence data stems from the 60s, 70s and 80s, so is based on paper and thin-layer chromatography (TLC) (49), photometric characterization of nucleosides following chromatography (50), and mass-spectrometry using low-resolution, low-sensitivity instruments (49). These methods failed to identify or distinguish some of the modifications, which are therefore listed as N and xN in Supplementary Table S1A but can now be detected with high-resolution mass spectrometry (51–54).

**Figure 1.**
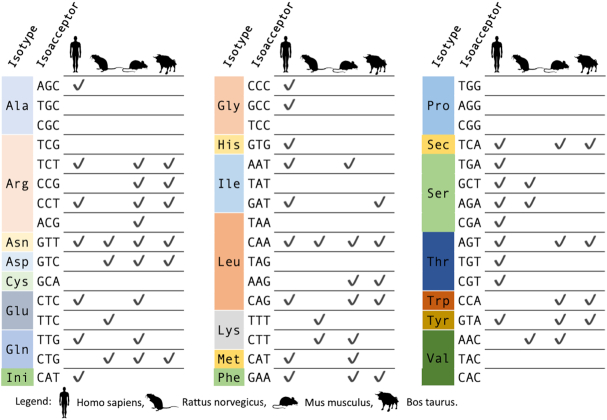
tRNA isoacceptors that have been biochemically characterized at the RNA level by traditional methods. Three additional tRNA isoacceptors (Rn-Val-NAC, Hs-Leu-NAA, Hs-Val-NAC) listed in Supplementary Table S1A weren’t placed in this figure due to their unknown nucleotide.

To add to the complexity of the task, the human genome (in contrast to yeast) encodes isoacceptor families that include many unique isodecoders, which are tRNAs with the same anticodon but contains variations in the tRNA body (42). Different isodecoders can be expressed under specific conditions as shown for the neuron-specific tRNA-Arg-UCU (55) or in the case of cancer (56). New high-throughput sequencing methods have been developed and optimized to facilitate detection of full length tRNAs such as DM-tRNA-seq (31) or tRNA-HydroSeq (57) or AlkAniline-Seq (58) and of tRNA-derived small RNAs (ARM-seq (32)). While these high-throughput RNA modification mapping methods reviewed in (59) and (30) are not yet as precise or quantitative as mass spectrometry, they do offer a practical, inexpensive method to survey a subset of modifications across all expressed tRNAs for many cell types. Using these methods also offers a first glimpse of the diversity of modification states across different isodecoders. Some isodecoder families in human such as Ala-AGC can be highly complex, contrasting the relatively simple view previously seen in budding yeast. In human, there are 22 high confidence Ala-AGC tRNA genes detected in the genome, which encode 16 unique (by sequence) Ala-AGC tRNA transcripts; in yeast, there are 11 Ala-AGC genes, which all encode identical Ala-AGC tRNA transcripts. This variation in human tRNA sequences also leads to an apparent complexity in tRNA modifications that is only now being appreciated. For example, RNA modification data collected with traditional methods exists for just 2 out of 16 Ala-AGC isodecoders (Ala-AGC-8 and Ala-AGC-11). ARM-seq and DM-tRNA-seq, however, both detect transcripts and modifications for many more isodecoders (Supplementary Table S5). These high-throughput methods allowed to detect four human modifications that were missing in MODOMICS at the time of our first analysis (see methods section). The final count of tRNA isoacceptors with modification information is 27 in humans and 38 in mammals (Figure 1 and Supplementary Tables S1A and S5).

### Linking the modifications of human cytosolic tRNAs to their corresponding modification enzymes

**Figure 2.**
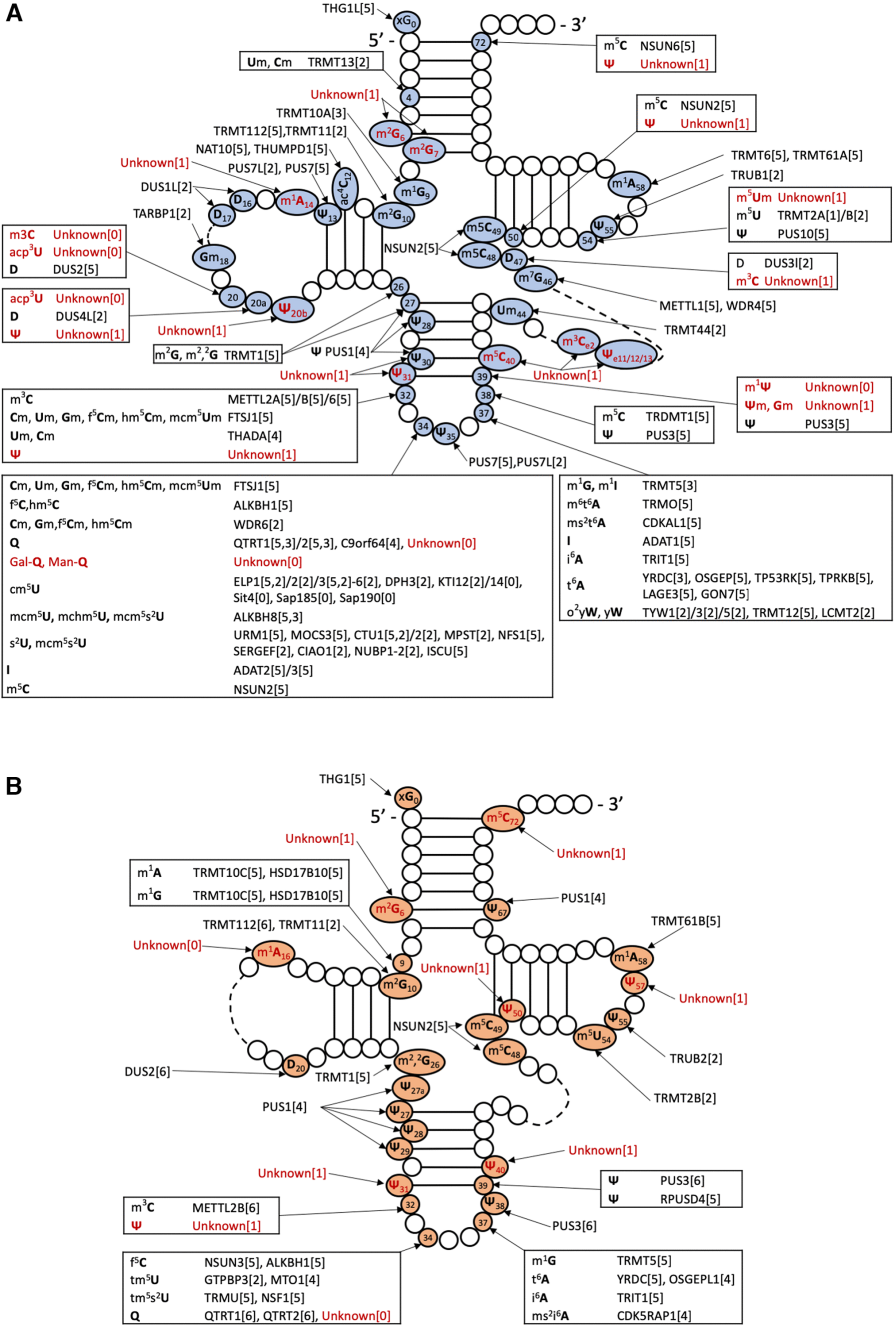
Cloverleaf representation of tRNA, with modified positions indicated for (**A**) cytoplasmic and (**B**) mitochondrial tRNAs, respectively, indicating genes/proteins experimentally validated in human, predicted with high confidence in other species, unknown with predictions, and unknown with no predictions.

We generated a current list of chemical modifications found in human cytosolic tRNAs (Figure 2A, Supplementary Table S2) by combining the modification information from the tRNA sequences compiled in Supplementary Table S1A Subsequently, we used this list as a starting point to generate the set of predicted human tRNA modification genes.

Once the list of human cytosolic tRNA modifications had been generated (Figure 2A, Supplementary Table S2), we linked the modifications to their corresponding modification enzymes whenever possible. This was done by using the advanced query tools of Uniprot for a first pass and then surveying the literature. By default, the reference linking the gene to the function is found by accessing the Uniprot entry for a given gene. Only when the reference had not yet been captured in Uniprot (∼10 cases), did we add a PMID entry in Supplementary Table S2.

Not all predictions reach the same level of credibility. For example, in some cases, experimental validation is available for the human ortholog, while in other cases only the function of the yeast ortholog is validated. Therefore, we used the following code to classify the evidence of our functional annotation: [5] *in vivo* data in mammals; [4] *in vivo* data of the human or a related mammalian enzyme in a heterologous host; [3] *in vitro* data using the human enzyme; [2] similarity to an experimentally validated gene in a non-mammalian species; [1] candidates that have not been verified in any organism; [0] no clear candidate. These predictions are available in Supplementary Table S2, and we summarized all enzymes with evidence codes 2–5 in Figure 2A, using the protein names recommended by the HUGO Gene Nomenclature committee (35). According to this assessment, we predicted at least 76 proteins to be required for the modification of cytosolic tRNAs. Clearly, this is an underestimation, as more than 24 enzymes are still unknown (evidence code 0 or 1). Furthermore, for approximately 26 proteins there is no direct *in vivo* or *in vitro* experimental data using a mammalian homolog (evidence code 2). Thus, our analysis emphasizes that extensive experimental validations and research will be required to verify specific gene predictions and to identify some of the ‘missing’ genes. These cases will be further discussed below.

### Identifying candidates for the ‘missing’ genes

To identify candidates of unidentified tRNA modifications enzymes, we compiled an initial list of ∼40 human proteins that are members of families known to be implicated in RNA modifications. These were mainly methyltransferases, pseudouridine synthases or THUMP-domain-containing proteins that have not yet been linked to a specific function. When we surveyed the recent literature, we found that ∼2/3 of these candidates had been reported to modify rRNA or proteins. For the remaining twelve proteins/genes, we gathered localization data from Mitocarta, and analyzed co-expression using the SEEK expression database profiles to identify the candidates that are coexpressed with RNA processing or translation related genes (Supplementary Table S4). This list is far from exhaustive as new methylase folds implicated in RNA modification are still being discovered (60).

### Missing genes coding for cytosolic tRNA modification enzymes

In general, when the gene is missing, the function of the corresponding modification is very difficult to infer, as no genetic study can be conducted. In some cases, such as acp^3^U20, the gene is not known in any organism, and almost no functional information is available. The only functional inference that can be done is if the gene encoding the enzyme responsible for the same modification is known in another organism. This is the case for a few modifications such as m^1^A14 and m^5^U54 in yeast or m^1^G6 in bacteria and archaea. Also, for complex pathways in which some genes have already been characterized such as Q and mcm^5^s^2^U34, functional information is available. However, we feel it is a far stretch to transfer functional inferences made from prokaryotes or unicellular eukaryotes to human. Even if a related enzyme is known in another species, it is very difficult to predict how the unknown human enzyme discriminates substrate tRNAs from non-substrates. Thus, in the absence of information about the gene and enzyme responsible, very little information can be inferred about the function of the modification itself. Below we list modifications of human cytosolic tRNAs, for which the genes remain to be discovered and characterized, and this list also indicates the areas where functional information is missing.

- **m^1^G6/7**: This nucleotide is modified in multiple cytosolic and in at least one mitochondrial tRNA (Supplementary Table S1A and B). Trm14/TrmN are members of the COG0116 family of methyltransferases and target this position in several thermophilic bacteria and archaea (61). However other members of the same family, such as RmlL have been shown to methylate guanine residues in 23S RNA (62). THUMPD2 and THUMPD3 are two barely characterized members of this family in humans (Supplementary Table S4), and previous analyses suggested that these enzymes might be required for the formation of both cytosolic and mitochondrial modifications (23). THUMPD2 was found to form a complex with the m^2,2^G26-methylase TRMT1, while THUMPD3 was shown to interact with the methylase-activator protein TRM112 (63) in two high-throughput interactome studies (36), strengthening their role as tRNA methyltransferase candidates. However, experimental verification will be required to evaluate whether these two proteins are essential for the formation of m^1^G, whether they exhibit different substrates specificities towards G6 or G7 and whether they act in mitochondria or in the cytoplasm.
- **m^1^A14**: Enzymes responsible for this modification were identified in *S. cerevisiae* and belong to the pfam01746 family (64). The human genome encodes three members of this family: TRMT10A is required for the generation of m^1^G9 in cytosolic tRNAs (65). TRMT10C as part of the RNase P complex, forms m^1^G9 in mitochondrial tRNAs (62) and like some family members from other species, can also methylate adenosine to form m^1^A9 (66). Hence, TRMT10B (Supplementary Table S4) is a candidate for the elusive m^1^A14 methyltransferase, even if a recent report could not detect any tRNA methylation activity *in vitro* (67). As expected for a cytosolic enzyme, TRMT10B is not part of the predicted human mitoproteome (37). However, multiple reports of interactions with 25 mitochondrial ribosomal proteins (https://thebiogrid.org/127659) suggest this protein localizes to the mitochondria. Further experiments will be needed to determine whether TRMT10B is the missing m^1^A14 methyltransferase or whether TRMT10A methylates both G9 and A14 or whether a yet unknown enzyme catalyzes this reaction.
- **Acp^3^U20,20a**: Only very few enzymes have been characterized that modify RNA by transferring the aminocarboxypropyl (acp) group of SAM, which is the methyl donor in most RNA-methylation reactions. However, acp-transferring enzymes belong to three unrelated superfamilies, which also contain methyltransferases. The only human enzyme currently known to introduce the acp^3^ modification is TSR3, a member of the COG2042 family (68), which is required for the biosynthesis of the hypermodified nucleotide m^1^acp^3^Ψ in 18S rRNA (69). The crystal structures of its archaeal homologs revealed that TSR3 belongs to the SPOUT class of methyltransferases (69). The second structurally characterized acp-transferase Tyw2 belongs to the unrelated RFM superfamily (70). A different acp modification has been described in the diphthamide-biosynthesis pathway, where an acp group is transferred from SAM to the carbon atom of a histidine residue of eukaryotic translation elongation factor 2 (eEF2) by an enzyme that belongs to the Radical-SAM superfamily (71). acp^3^U is found in several positions in tRNA of different organisms like for example acp^3^U47 in *E. coli* tRNA, but the corresponding enzymes have not been identified in any of these species. Since all known acp transferases most likely arose independently from methyltransferases, the acp^3^U-forming enzyme may currently be annotated as a hypothetical methyltransferase of unknown function (Supplementary Table S4) but it is difficult to select a plausible candidate in light of the diversity of known acp transferases.
- **Ψ**: The list of pseudouridine synthases modifying human tRNAs is far from complete. Several candidates have been proposed to be required for the modification of positions 30–32, 50, 72 or e11,12,13 (23), but several can likely be excluded as they were found to be required for the modification of mitochondrial rRNA and mito-tRNA at positions 27, 29, 39 and 50 (RPUSD4) (65,72) or mitochondrial mRNA (like RPUSD3) (73). RPUSD1 and RPUSD2 (Supplementary Table S4) have not been tested experimentally and are hence still valid candidates. Pus7/TruD, the enzyme that introduces Ψ13 is highly conserved in all three kingdoms (74) and is a member of the COG0585 family. The yeast Pus7 enzyme further modifies position 35 (75). PUS7 and PUS7L, two members of the COG0585 family in humans are products of a gene duplication that occurred most certainly in the common ancestors of metazoa (see http://phylomedb.org/?q=search_tree&seqid=Q9H0K6). Experiments will be required to determine whether these two enzymes have identical, overlapping or different substrates specificities. For example, one of the two enzymes might modify position 13, while the second enzyme might target position 35. Another possibility is that PUS7 and PUS7L target both positions 13 and 35, but in different tRNA isoacceptors. PUS7 is implicated in pseudouridylation of Ψ8 in tRF derived from Ala-tRNA, Cys-tRNA and Val-tRNA but whether PUS7 acts directly on tRNA has formally not been shown (76). PUS1 is multisite specific so it is a plausible candidate for the positions 30 to 32, even though it has been found that the mouse homolog modifies positions 27, 28, 34 and 36 (77). Finally, based on experimental evidence from Archaea (78), it had been postulated that the human Pus10 is required for the formation of Ψ54 (28) and this was recently experimental validated in human (79).
- **Q34**: Queuosine in position 34 (Q34) is highly conserved in bacteria and eukarya. Humans like all eukaryotes are unable to synthesize Q but instead salvage the queuine (q) base from their diet and gut microflora as a micronutrient (80). Recent studies have shown that nutritionally determined Q-tRNA levels promote Dnmt2-mediated methylation of tRNA-Asp and control translational speed of Q-decoded codons as well as at near-cognate codons (81). The heterodimeric human TGT enzyme formed by the QTRT1 and QTRT2 (previously called QTRTD1) subunits is the only fully characterized enzyme of the Q salvage pathway (80). A second human salvage-enzyme member of the DUF2419 family has been identified but its molecular function is unknown (82). Finally, the transporter for the q base or the precursor nucleoside Q is still elusive as well as the enzyme(s) that further modify the Q residue by attaching galactosyl or mannosyl moieties.
- **mcm^5^s^2^U34**: Wobble uridine is generally modified in all known organisms (see (83) for a recent review). The combination of modifications at positions 2 and 5 of the nucleobase results in an intricate tuning of codon-anticodon interactions, thus allowing the translation apparatus to distinguish codons in split-codon boxes and to introduce additional amino acids (83,84). 5-carboxymethyluridine (cm^5^U), the first step of the 5-modification is introduced by the action of the Elongator complex, a heteromeric complex consisting of two copies of Elp1–Elp6 that is activated by several auxiliary proteins (85). Orthologs of all yeast Elongator complex subunits are known and described in humans. However, human orthologs of the yeast regulatory components (the kinase Kti14, the phosphatase Sit4 and its regulatory subunits Sap185 and Sap190) could not be identified. Here, functional screens will be required to determine the counterparts of these components in humans. The conversion of cm^5^U to mcm^5^U is catalyzed by the c-terminal Trm9 domain of ALKBH8 (86–88). mcm^5^U in some tRNA can be further hydroxylated to mchm^5^U by the AlkB Domain of ALKBH8 (87) or *2*′-*O*-methylated to mcm^5^Um by an unknown enzyme. The enzyme required for ncm^5^U formation from cm^5^U is not known in any organism and remains to be identified. 2-thiolation is achieved through the action of the URM1 pathway that shares features of bacterial sulfur-carrier proteins (SCP) and ubiquitin-like proteins (UBL) (89). The URM1 pathway components are straight forward to identify. Urm1 has two homologs in humans: URM1 and MOCS2A. However, MOCS2A is required for the synthesis of the molybdopterin cofactor while URM1 is required for tRNA thiolation and MOCS3 activates the SCP of both pathways (90). The final step of the thiolation reaction is performed by a complex consisting of CTU1 and CTU2.
- **Missing methyltransferases**. Methyltransferases are the biggest group of RNA modifying enzymes. While many tRNA methyltransferases have been discovered and characterized, a few of them remain to be identified (Supplementary Table S2). Members of the NSUN family (PF01189) usually introduce m^5^C modifications (91) and some such as NSUN2 are multi-site specific (92). However, NSUN2 is not required for the formation of m^5^C40 or m^5^C72 (92). NSUN7 is the only member of the NSUN family without a known substrate (Supplementary Table S4). Hence, it is a strong candidate for methylating one or both these positions. However, indirect data links it to methylation of enhancer RNAs (93). Three enzymes (METTL2A, METTL2B and METTL6) have been found to be involved in m^3^C32 formation potentially on different tRNA targets (94). Any of these three might be required for introducing m^3^C at position e2 and/or 47 as the biochemical assays have been inconclusive to date (See (94), Supplementary Table S5). It is unclear, which protein synthesizes m^5^U54 since two human homologs of yeast Trm2 were identified: TRMT2A and TRMT2B (Supplementary Table S2). It is not known whether these two proteins catalyze the same reaction or whether they differ in substrate specificity or sub-cellular localization. For example, TRMT2B is predicted to localize to mitochondria and might be required for modifying mitochondrial tRNAs (Supplementary Table S3). Finally, no candidate can easily be proposed for the formation of m^1^Ψ39, Ψm39 and Gm39. The pool of methyltransferase candidates among proteins with uncharacterized functions is large (∼8, Supplementary Table S4), and we did not find evidence to favor a specific candidate.

### Identification of the genes encoding for mitochondrial tRNA modifications enzymes

The Suzuki laboratory published a thorough compilation of tRNA modification enzymes for the full set of 22 bovine mito-tRNAs (23) and most of their functional annotations can be transferred to orthologous human enzymes (Figure 2B, Supplementary Table S3). Furthermore, some open cases have been solved since. Notably, ALKBH1 and NSUN3 are required for the formation of f^5^C in initiator tRNA (95–97). The same ALKBH1 enzyme is further required for hm^5^C and f^5^C formation in cytosolic tRNA (95). A more complete compilation of the predicted human mitochondrial tRNA modification enzymes was published recently with extensive added functional information (24). We compiled these predictions and added evidence codes resulting in a list of 35 enzymes required to modify the full set of mitochondrial tRNAs (Figure 2B and Supplementary Table S3). An additional evidence code to classify enzymes that have been experimentally validated in the cytoplasm but not in mitochondria was added (evidence code 6). We will discuss here the remaining open questions.

The Q base is found in mitochondrial tRNAs and the catalytic subunit QTRT1 of the human transglycosylase complex is found in the mitoproteome (Supplementary Table S3). In the cytoplasm, QTRT1 forms a complex with QTRT2 (98) but it is not known whether this interaction also occurs in mitochondria. It has been shown that QTRT1 and QTRT2 are associated with the mitochondria with QTRT2 more loosely bound than QTRT1 (99). Is it possible that QTRT2 facilitates the transport of q, as the mitochondrial queuine transporter is missing?

Similar to cytosolic pseudourine synthases, the set of enzymes introducing Ψ residues in mitochondrial tRNAs is far from complete, in particular since different enzymes can introduce the same modification at a given position in different tRNAs, implying that many more might be missing. RPUSD4 was recently shown to modify 16S rRNA from mitochondria and introduce Ψ39 in mito-tRNA^Phe^ but not in mito-tRNA^Gly^ (72). PUS3 was predicted to modify other mitochondrial tRNAs such as mito-tRNA^Gln^ at position 39 (23). However, experimental data on PUS3 is available only for cytosolic tRNAs, requiring additional confirmation of its mitochondrial targets. Two pseudouridine synthases without known substrates (RPUSD3 and PUS1L) localize to the mitochondria (Supplementary Table S4). RUPSD3 modifies mitochondrial mRNAs (73), leaving PUS1L as a strong candidate for an enzyme that modifies positions 30, 31, 50 and/or 57 (Supplementary Table S4). Nevertheless, we cannot exclude that a pseudouridine synthase not predicted to be mitochondrial such as RPUSD1 or RPSUD2 is actually dually-targeted as it has been recently shown for Pus10 that is translocated to the mitochondria only under specific physiological conditions (100).

In general, the situation is more complex when one gene encodes for two proteins that localize to different sub-cellular compartments, since the mitochondria-targeted isoform is often not identified as a mitochondrial protein. Thirty- seven proteins are predicted to be required for mitochondrial tRNA modifications with nine of these currently unknown, and eleven modify only mitochondrial tRNAs (Supplementary Table S3). In the Mitocarta analysis that integrates 14 different sources of predictions and experimental data to compile a list 1158 human mitochondrial protein (37), ten of these proteins were correctly identified as mitochondrial (Supplementary Table S3). The only exception is CDK5RAP1, an enzyme required for the thiolation reaction during ms^2^i^6^A formation (101,102). Of the dually targeted proteins, ten were correctly assigned as mitochondrial in Mitocarta (Supplementary Table S3) while seven others were not: TRM5, YRDC, PUS3, NSUN2, TRM112, METTL2B and QTRT2. TRM112 and QTRT2 are non-catalytic subunits and it cannot be excluded that they are dispensable in mitochondria, as tRNA modification machineries can be simpler in mitochondria. For example, only two proteins are required for the synthesis of t^6^A in mitochondria while six proteins are required in the cytosol (103). The case of PUS3 has already been discussed above. For the remaining three cases, dual localization data in yeast for Sua5p (104) and for Trm5p (105) as well as predicted isoforms (see http://www.uniprot.org/uniprot/Q08J23 for NSUN2) suggest that they can be similarly found in both compartments in humans. Hence, the MitoCarta set of mitochondrial proteins may be incomplete.

Finally, the enzymes required for the formation of m^2^G6, m^5^C72 and of several Ψ residues are experimentally uncharacterized (Supplementary Table S3). As discussed above, the two candidates for the formation of cytosolic m^2^G6 are THUMPD2 and THUMPD3. These two proteins are not predicted to localize to mitochondria (37) but this prediction might not be correct. The same is true for the candidate for the cytosolic m^5^C72 methylase, NSUN7.

### Tissue expression and proteomics data of tRNA modification enzymes

**Figure 3.**
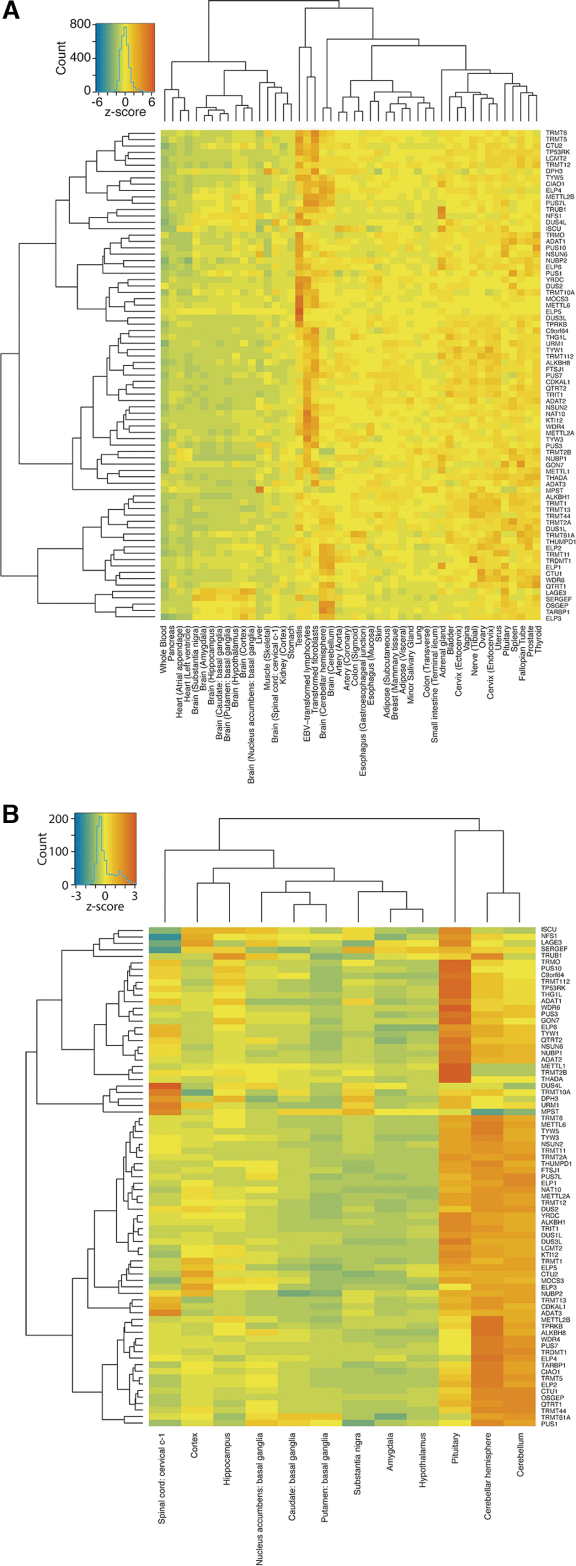
Expression of tRNA modification genes and candidates in a representative set of healthy human tissues. (**A**) Empirically selected tissues with physiological relevance are included. (**B**) Like (A), but only representing a set of brain tissues. Genes are clustered according to similarity in the expression profile. Source: gtexportal.org. Genes included are from Supplementary Table S2.

Disease phenotypes of aberrant tRNA modification enzymes are often linked to neuronal phenotypes, metabolic disorders and cancer (17–21). However, the tissue-specific expression profiles of tRNA modification genes have not been systematically explored. Therefore, we used expression data available through the Genotype-Tissue-Expression (GTEx) project and compared expression levels of all modification genes in representative tissues of all organs (Figure 3). We also compiled tissue-specific proteomics evidence from the human proteome map (41) (Supplementary Figure S1). Overall, there are several general trends: First, expression levels of modification genes are quite uniform between different tissues. Second, expression levels of most genes are relatively low, in particular in whole blood. Third, generally high expression levels are observed in testis and transformed cells. Fourth, expression levels in brain are below average of the tissues with the exception of the cerebellum, where expression of a small number of genes reaches levels that are similar to expression observed in testis. This is surprising given the typical neuronal phenotypes observed upon defects in human modification enzymes. There are, however, clusters of genes that are upregulated in several brain tissues (Figure 3). These findings may point to a more crucial function during early steps of differentiation and proliferation of stem cells. Thus, it is likely that many of the observed defects in humans are either developmental phenotypes or relatively subtle. Finally, even though most genes of different pathways are found in similar clusters, this is not true for all pathways, e.g. the ELP pathway. A similar trend is seen when analyzing the proteomics data (Supplementary Figure S1). With the highly expressed genes, a correlation was observed between the transcriptomic and proteomics data (for example *DUS2, PUS3* or *TRMT5* in testis), however, the proteomic data is less complete due to the low expression levels of most enzymes (Supplementary Figure S1). These observations suggest that tRNA modification enzymes are able to maintain sufficiently high modification levels in differentiated tissues likely because of the high stability of tRNA and low tRNA synthesis levels. Furthermore, some modification enzymes bind to RNA that are not their natural targets (See (106–109) for specific examples). Hence, it is likely beneficial to maintain low expression levels of these enzymes to avoid unspecific modification of cellular RNA like mRNA or rRNA.

## CONCLUSIONS

This inventory of human tRNA modifications and the corresponding enzymes surprisingly reveals that despite the fact that the field of RNA modifications has dramatically expanded in recent years with 50 human modifications enzymes identified only in the last 10 years, the picture is far from complete. We estimate that between ∼135 genes are required to modify cytosolic and mitochondrial tRNAs and that 23% of these genes still need to be identified and that another 22% require further experimental validations. Approximately 50% of the human modification genes have been linked to a number of human diseases (Table 1). Furthermore, all genes required for the formation of iron-sulfur clusters affect tRNA modification indirectly and are linked to diseases that are likely not mediated by tRNA modification defects. Like described before (17–22) the phenotypes are most often neurodegenerative or neurodevelopmental diseases like microcephaly and intellectual disabilities, but also renal and metabolic defects. Finally, roughly 50% of the disease genes have been linked to cancer (Table 1), suggesting that tRNA modification enzymes may provide interesting targets for cancer therapies. Also, given the wide diversity of tRNA transcript sequences in humans, the preference of different members of the modification enzyme families for different tRNA isodecoders remains an open question. An in-depth analysis of tRNA modification dynamics in various stress conditions and cell types will reveal the intimate relationship between tRNAs and their modifying partners in more detail. This compilation can act as a guide for future experiments to complete the characterization of the set of human tRNA modification enzymes.

## Supplementary Material

Supplementary Data
