# Supplementary material for: Matching tRNA modifications in humans to their known and predicted enzymes
Source: Nucleic Acids Res. 2019 Jan 30;47(5):2143–59. doi: 10.1093/nar/gkz011 (PMC6412123; doi:10.1093/nar/gkz011)
Supplement: Supplementary Data [file gkz011_supplemental_files.zip › Supplementary material legends.pdf]

SUPPLEMENTARY DATA

Table S1 Compilation of modifications and their positions in different cytosolic (A) or mitochondrial (B) mammalian tRNAs

Table S2 List of tRNA modifications with predicted corresponding enzymes for cytoplasm

Table S3 List of tRNA modification with predicted corresponding enzymes for mitochondria

Table S4 List of potential candidates for missing tRNA modification enzymes

Table S5 List of predicted human tRNA modifications using new, high-throughput sequencing methods (29,30,57). Modification data shown is from these same studies (29,57).

Figure S1. Protein level across different tissues according to www.humanproteomemap.org. The color key indicates the normalized spectral counts per proteins detected across the tissues using only unique peptides (red = 1; white = 0).
