# Supplementary figures and images for: Matching tRNA modifications in humans to their known and predicted enzymes
Source: Nucleic Acids Res. 2019 Jan 30;47(5):2143–59. doi: 10.1093/nar/gkz011 (PMC6412123; doi:10.1093/nar/gkz011)

Gene

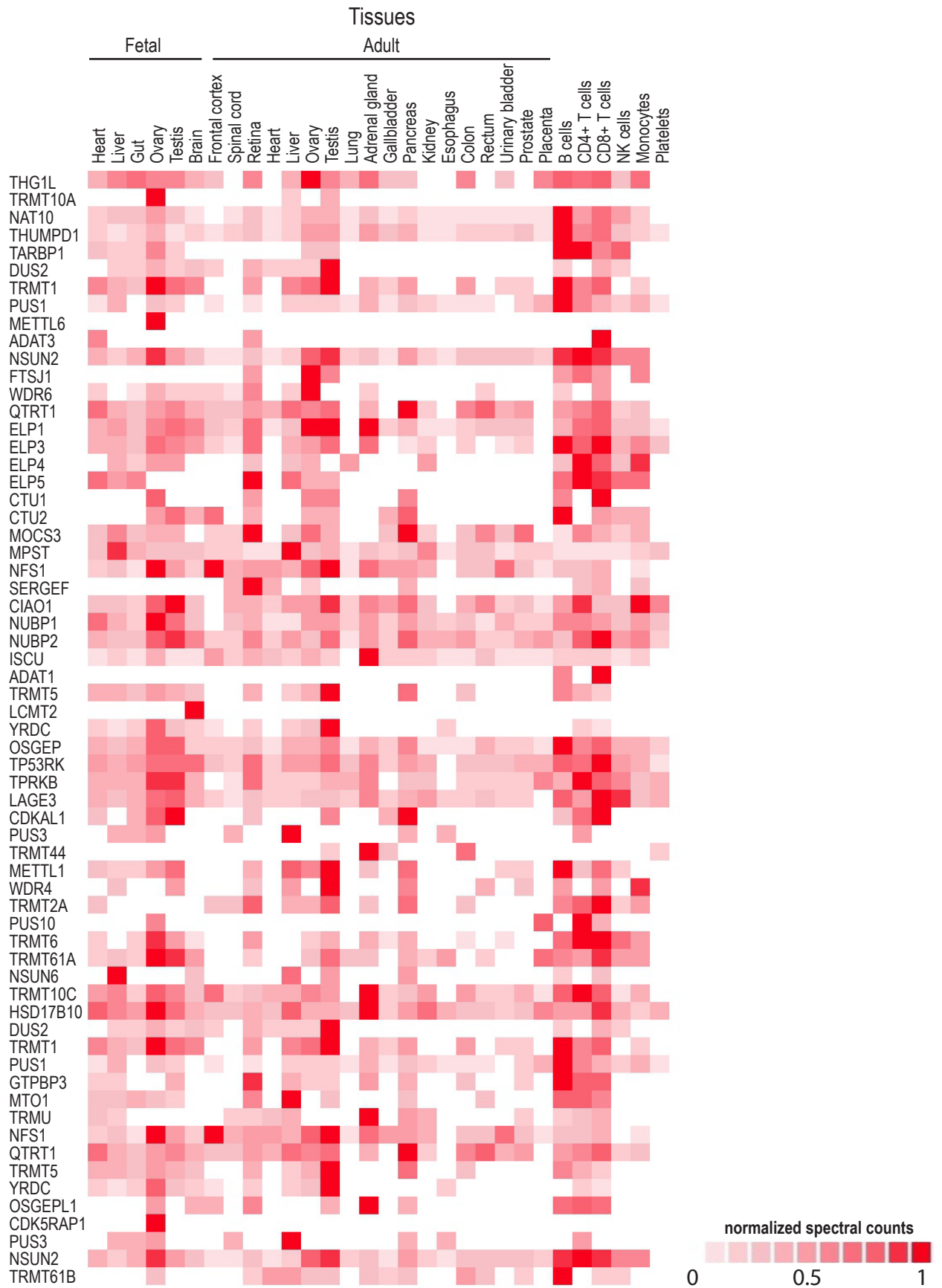

Supplement: Supplementary Data [file gkz011_supplemental_files.zip › Figure_S1.pdf]
